# Responding to a Tragedy: Evaluation of a Postvention Protocol Among Adult Psychiatry Residents

**DOI:** 10.1007/s40596-021-01418-x

**Published:** 2021-03-08

**Authors:** Alpna Agrawal, Michael Gitlin, Sir Norman T. Melancon, Brittany Irshay Booth, Jennifer Ghandhi, Katrina DeBonis

**Affiliations:** Los Angeles Medical Center, Los Angeles, CA USA

**Keywords:** Postvention, Adverse events, Physician, Burnout, Perceived growth

## Abstract

**Objective:**

In a time of “zero suicide” initiatives and rising suicide rates, resident physicians are particularly susceptible to the psychological and professional ramifications of patient suicide. An adult psychiatry residency program developed and implemented a postvention protocol to address the impact of patient suicide among resident physicians. The current study is a formal evaluation of a training program’s postvention protocol from June 2018 to April 2020.

**Methods:**

Process and outcome indicators were identified to assess protocol implementation and effectiveness. Process indicators included were postvention protocol adherence. Outcome indicators were perceived helpfulness of postvention protocol–related supports, occupational and general health measures, posttraumatic growth, and posttraumatic stress symptoms following resident participation in the postvention protocol.

**Results:**

Study response rate was 97% (*n* = 57/59) and 81% completed the entire survey (*n* = 48/59). Twenty percent of residents (*n* = 10/48) experienced patient suicide during residency. Postvention protocol adherence was between 57 and 100%. Protocol-related supports, such as speaking with attendings who had previously experienced an adverse event, were more helpful than other supports (*p* < 0.01). Compared to residents who had not experienced patient suicide, mean work empowerment, burnout, mental health, and quality of life scores were not significantly different from residents who participated in the postvention protocol (*p* > 0.05). Posttraumatic growth was positively correlated with self-determination at work (*p* = 0.01).

**Conclusions:**

The postvention protocol was helpful to residents and potentially effective at mitigating the psychological and professional consequences of patient suicide. Study findings may inform standardization of postvention protocols among psychiatry training programs.

In the field of psychiatry, there is ongoing debate regarding the predictability and preventability of suicide. “Zero suicide” campaigns and the lack of clinical prediction tools illustrate a gap between the field’s aspirational goals and observed outcomes. In an era of initiatives to eradicate suicide, psychiatrists, particularly trainees, may be even more susceptible to the psychological and professional distress of patient suicide [1].

Psychiatrists experience higher rates of patient suicide during training than later in their career [2–4]. In one study, twenty percent of residents reported more than one patient suicide [3]. Residents are at high risk to be traumatized and report changes in their professional practice following patient suicide. They report problems with clinical decision-making, hospitalize patients more frequently, and may consider a career change [5–7]. Given their less well-formed professional identity, trainees may find it particularly difficult to ask for help. In one study, one-quarter of residents were unable to ask for support after patient suicide, though they knew help was available to them [3].

Despite the prevalence of patient suicide during training, there remains no mandate from organizations such as the American Association of Directors of Psychiatry Residency Training (AADPRT) or the Accreditation Council for Graduate Medical Education (ACGME) to require psychiatry training programs to provide formal education in this area [8, 9]. An ideal curriculum for a training program would include an educational component that all residents would receive to prepare them for the loss of a patient to suicide and an “as-needed” or postvention response that is clearly outlined and implemented when the program or resident learns of the death of a patient to suicide (or even a near-fatal suicide attempt). A national survey of psychiatry chief residents and program directors showed that only one in five training programs had a postvention protocol in place [10].

In response to this training gap in residency education, an adult psychiatry residency program director developed and implemented a postvention protocol for residents in June 2018. Based on leading residents through difficult patient outcomes, the program director recognized that patient suicide and related fears can trigger acute stress symptoms in trainees. The postvention protocol was created as a mechanism to help residents cope with these events using knowledge of how resiliency is cultivated [11]. Recognizing that a metaphorical “potential space” exists between a trainee’s experience of loss following patient suicide and a training program’s role in shaping how this experience is integrated into the trainee’s professional identity, the postvention protocol sought to facilitate resiliency and growth following such a tragedy in an effort to fill this “potential space.” The postvention protocol focused on minimizing the negative impact of patient suicide on trainees’ mental health and professional identity [12]. While postvention protocols have been proposed in the literature, none has been formally evaluated among early career physicians [13–16]. The current study evaluated the postvention protocol among adult psychiatry residents who participated in the protocol from June 2018 to April 2020. Process and outcome indicators were identified to assess protocol implementation and effectiveness. The study is unique given its application of validated measures not previously examined among physicians reporting adverse events.

## Methods

### Postvention protocol description

**Fig. 1:**
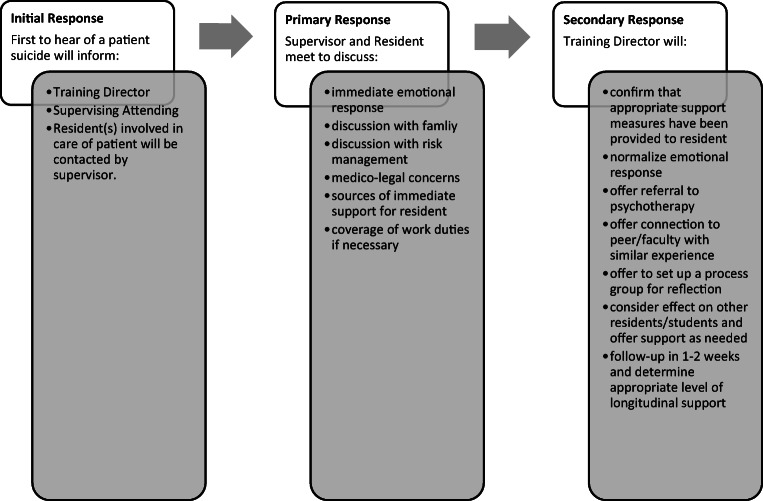
Postvention protocol developed by an adult psychiatry residency training program, June 2018

The protocol provides the necessary support and guidance to help individual trainees cope with the loss of a patient to suicide and potentially facilitate personal and professional growth. Via a three-tiered response system, the protocol identifies initial, primary, and secondary response stages (Fig. 1). The initial response stage includes rapid notification of team members and family regarding patient suicide. Based on previous research, the initial response stage is purposefully delineated from the primary response stage which involves an intentional discussion between the supervisor and resident regarding the trainee’s immediate emotional response and practical concerns following patient suicide. In the primary response stage, the supervisor and resident debrief and process the event together, thereby reducing feelings of isolation and normalizing the resident’s anticipated emotional response. The primary response point of contact also provides an opportunity to determine who would be the most appropriate person to contact the family and speak with the risk management team, if necessary.

The secondary response, overseen by a training director, provides a mechanism for ensuring that the primary response was appropriately conducted and subsequently to offer other sources of support to promote recovery and healing such as individual therapy, the convening of a process group, or connection to faculty member or peer who has been through a similar experience. A training director will also consider the response of other residents in the program who may be affected indirectly by knowledge of the patient suicide. Finally, the training director or supervising attending follows up with the trainee over time at mutually agreed upon intervals, usually within a week or two of the event, with continued follow-up based on the needs of the resident. Follow-up meetings by phone or in person served to monitor the psychological and professional impacts of the event and provide the appropriate level of ongoing support. The majority of residents indicated that after the second follow-up session, they felt confident that they were in the process of recovering from the event and did not feel the need for continued scheduled follow-up.

### Postvention protocol evaluation design

Process indicators capture whether a program’s components are successfully implemented, and outcome indicators measure whether a program’s objectives were achieved [17]. Process indicators in this study assessed whether the postvention protocol steps described at each response stage occurred. Outcome indicators selected were based on the described impact of patient suicide on trainees in the literature [3, 18–21]. Indicators examined were [1] the helpfulness of supports offered, [2] occupational and general health outcomes, and [3] posttraumatic growth and posttraumatic stress disorder (PTSD) symptoms.

### Study design

The study sample was a part of the Physician Work and Health study initiated in June 2018 by a fourth year resident, the principal investigator, and adult psychiatry residency program director. The study’s primary purpose was to assess the program’s educational and training priorities. The current study was part of this larger project examining occupational and general health outcomes among physicians. The survey instrument for the larger project was administered via the Qualtrics® platform at two time points in October 2019 and April 2020. At the second time point, an Adverse Event Module was included to evaluate the program’s postvention protocol. Study participation was voluntary and anonymous. Residents were incentivized to participate by gift card raffles and the provision of food and refreshments when possible. The UCLA institutional review board (IRB) approved the study. The IRB waived signed informed consent and approved an information sheet provided to participants prior to initiating the survey.

### Study sample

The current study examined data from the Physician Work and Health survey implemented at time point 2. A total of 57 out of 59 eligible residents participated in the survey yielding a response rate of 97%. Forty-eight subjects completed the entire survey (81% of eligible residents) and nine, partially completed the survey.

### Postvention protocol process indicators

#### Postvention protocol adherence

Residents provided yes/no responses regarding whether processes a part of each stage of the postvention protocol occurred. For example, residents were asked if contact with the supervising attending occurred immediately following the event (e.g., initial response stage), if work modifications were discussed (e.g., primary response stage), and whether mental health services were offered (e.g., secondary response stage).

### Postvention protocol outcome indicators

#### Helpfulness of support offered

Residents were asked whether supports they utilized that did and did not follow from the postvention protocol were helpful on a 5-point Likert scale. The “protocol-related support” index included six items related to support residents received from supervisors and risk management. For example, residents were asked how helpful it was to speak with a faculty member who had experienced an adverse event. The “other support” index included six items related to support residents received from co-workers and family. Indices were computed by summing item responses and dividing by the total number of items. Items created were consistent with previous research describing supports utilized by psychiatrists following patient suicide [22].

#### Occupational and general health measures

Work empowerment scale is a 12-item scale that measures one’s competence, meaning, self-determination, and impact at work on a 5-point Likert scale [23]. Higher scores indicate higher levels of empowerment. The Cronbach’s *α* for the scale was 0.88.

The Maslach Burnout Inventory for medical personnel is a 22-item scale that measures emotional exhaustion, depersonalization, and personal accomplishment at work [24]. A higher score on the emotional exhaustion and depersonalization scales and lower score on the personal accomplishment scale indicate higher occupational burnout. Cronbach’s *α* for the emotional exhaustion scale was 0.93; the depersonalization scale, 0.80; and the personal accomplishment scale, 0.84.

Mental health was assessed by the SF-12v1 [25]. Using norm-based scores, the SF-12v1 produces a composite score of self-reported mental health ranging from 0 (worst) to 100 (best). The scaled score enables comparison between study populations and US national averages [26]. Quality of life was measured by a single item asking respondents to rate their overall quality of life [27].

#### Posttraumatic growth

Posttraumatic growth was measured using the Posttraumatic Growth Inventory, a 21-item scale with 5 subscales: relating to others, new possibilities, personal strength, appreciation of life, and spiritual change [28]. Scale instructions were modified by asking residents to indicate change as a result of “their professional experience with patient suicide.” A higher score indicated higher posttraumatic growth. Cronbach’s *α* for the PTGI scale was 0.77.

#### PTSD symptoms

The impact of events scale–revised (IES-R) is composed of 22 items that assess PTSD symptoms following a stressful event by three subscales: intrusive thoughts, avoidance, and hypervigilance [29]. Scale instructions were modified by asking residents to recall their distress during the month following their patient suicide. Cronbach’s *α* for the IES-R scale was 0.89.

### Resident feedback

Residents were asked to provide open-ended feedback regarding the postvention protocol, specifically whether there was anything missing in their discussions with program administration and supervisors, and what would have been helpful at the time of and after their adverse event.

### Analysis

Study sample characteristics were reported by descriptive statistics. Indicator mean scores and proportions were compared with available values from similar populations in the literature. Statistical testing included two-sample *t* tests, two-sample proportion tests, and Pearson’s correlations. Stata 16.1 was used for analyses and *p* < 0.05 was considered statistically significant. Open-ended feedback from residents regarding the postvention protocol is displayed by exemplar quotes highlighting themes of feedback.

## Results

### Prevalence of patient suicide among adult psychiatry residents

Twenty percent (*n* = 10/48) of residents reported patient suicide (Table 1). Among interns, 8% (*n* = 1/13) experienced patient suicide 3–6 months before survey data were obtained. Twenty percent of second year residents (*n* = 3/14) reported patient suicide between 3 months and 2 years ago. Ten percent of third year residents (*n* = 1/10) experienced patient suicide 1 to 2 years ago. Forty percent of fourth year residents (*n* = 5/12) experienced patient suicide within the last 3 months to 2 years ago (Table 2). In other words, among residents reporting patient suicide (*n* = 10), half (*n* = 5/10) reported the event occurred during their first 2 years of training and the other half (*n* = 5/10), during their latter 2-year training.

**Table 1:** Descriptive characteristics of study sample by residents’ experience of patient suicide (*N* = 49), April 2020

	No patient suicide (*n* = 39)	Patient suicide (*n* = 10)
Characteristics	% (*n*)	% (*n*)
Total sample	80 (39)	20 (10)
Year of training		
Intern	92 (12)	8 (1)
Second year psychiatry resident	79 (11)	21 (3)
Third year psychiatry resident	90 (9)	10 (1)
Fourth year psychiatry resident	58 (7)	42 (5)
Time since patient suicide		
0–3 months ago	-	10 (1)
3–6 months ago	-	20 (2)
6–12 months ago	-	40 (4)
1–2 years ago	-	30 (3)

**Table 2:** Postvention protocol adherence (*n* = 9), April 2020

		Yes	No
Procedure	*n*	%	%
Initial response stage^1^			
Notification of supervisor	9	100	0
Primary response stage^1^			
Discussion about emotional impact	9	89	11
Work modifications discussed	9	56	44
Secondary response stage^1^			
Meeting with program director occurred	9	89	11
Mental health referral offered	9	67	33
Peer support offered	9	89	11
Follow-up 1–2 weeks by supervising attending	9	89	11

^1^Refer to postvention protocol (Fig. 1) for details

### Postvention protocol process indicators: procedure adherence

Of residents who completed the Adverse Event Module (*n* = 9), all underwent the postvention protocol. Residents indicated that five out of seven postvention protocol steps occurred a majority of the time (89–100%) (Table 2). Protocol processes with lower reported occurrence were discussion of work modifications (56%) and mental health referral (67%).

### Postvention protocol outcome indicators: helpfulness of postvention protocol supports

Residents were significantly more likely to report that protocol-related supports (mean index score = 3.32, SD = 0.19) were more helpful than other types of support (mean index score = 2.42, SD = 0.24) (*p* < 0.01). Protocol-related supports ranked highly were as follows: support from an attending who had experienced an adverse event, the program director, and supervising attending. Other supports that ranked highly were support from other supervising attendings and co-residents (Table 3).

**Table 3:** Helpfulness of supports utilized by residents following patient suicide (*n* = 9), April 2020

		Extremely helpful	Very helpful	Moderately helpful	Slightly helpful	Not helpful at all	
Type of support	*n*	%	%	%	%	%	Mean (SD)
Postvention protocol supports index score^1^	9						3.3 (0.6)
Program director	6	50	50	0	0	0	3.5 (0.5)
Supervising attending	9	44	44	11	0	0	3.3 (0.7)
Patient’s family	4	25	25	50	0	0	2.8 (1.0)
Attending who experienced adverse event	9	67	22	11	0	0	3.6 (0.7)
Mental health services	3	33	33	0	33	0	2.7 (1.5)
Risk management	3	0	33	0	67	0	1.7 (1.2)
Other supports index score^1^	9						2.4 (0.7)
Other supervising attending	6	33	50	17	0	0	3.2 (0.8)
Resident a part of patient’s care	3	0	33	67	0	0	2.3 (0.6)
Resident not a part of patient’s care	8	25	63	13	0	0	3.1 (0.6)
Friend not in medicine	6	0	33	17	17	33	1.5 (1.4)
Partner	9	22	0	44	33	0	2.1 (1.2)
Family	7	29	14	14	43	0	2.3 (1.4)

*SD* standard deviation

^1^Index score computed by summing item responses and dividing by the total number of items

### Postvention protocol outcome indicators: occupational and mental health outcomes

Compared to residents who had not experienced patient suicide, residents who participated in the postvention protocol were not significantly different on measures of mean work empowerment, burnout, mental health, and quality of life scores (*p* > 0.05, Table 4). Mean subscale scores for burnout did not differ between residents who had not experienced patient suicide and residents who had participated in the postvention protocol (*p* > 0.05, results not shown). Based on a prior report in the literature, mean emotional exhaustion score among physicians on a transplant team with a debriefing protocol (mean = 2.6, SD = 1.08) was similar to residents in this study who participated in the postvention protocol (mean = 2.5, SD = 1.05) (*p* > 0.05) [30].

**Table 4:** Outcome indicators among residents who had not experienced patient suicide and residents who reported patient suicide and participated in the postvention protocol (*N* = 49), April 2020

	No patient suicide			Patient suicide and postvention protocol				
Measures	*n*	Mean	SD	*n*	Mean	SD	*t* statistic	*p* value
Occupational health								
Work empowerment score	39	61.5	9.1	10	64.5	6.1	−1.09	0.29
Competence subscale score	39	5.4	0.8	10	5.6	0.7	−0.87	0.40
Self-determination subscale score	39	5.1	1.1	10	5.5	0.8	−0.97	0.35
Impact subscale score	39	4.0	1.3	10	4.4	1.2	−1.15	0.27
Meaning subscale score	39	0.5	0.83	10	5.9	0.6	0.35	0.73
Maslach burnout inventory score								
Emotional exhaustion subscale score	39	21.7	11.8	10	22.6	9.4	−0.37	0.71
Depersonalization subscale score	39	9.6	6.5	10	8.6	4.3	0.44	0.66
Personal accomplishment subscale score	39	38.0	6.2	10	39.4	6.4	−0.41	0.69
General health								
Mental health composite scale score, SF-12v1	39	39.9	12.9	10	44.2	9.1	−0.96	0.35
Quality of life	39	5.3	1.1	10	5.4	0.8	0.37	0.72
Posttraumatic growth								
Posttraumatic Growth Inventory score	-	-	-	9	18	7.2	-	-
Relating to others subscale score	-	-	-	9	7.4	4.5	-	-
New possibilities subscale score	-	-	-	9	1.4	1.1	-	-
Personal strength subscale score	-	-	-	9	4.0	2.5	-	-
Spiritual change subscale score	-	-	-	9	0.2	0.4	-	-
Appreciation of life subscale score	-	-	-	9	2.8	2.4	-	-
Posttraumatic stress disorder symptoms								
Impact of event scale-revised (IES-R) score	-	-	-	9	19.2	10.7	-	-
Intrusion subscale score	-	-	-	9	9.8	5.5	-	-
Avoidance subscale score	-	-	-	9	5.7	4.0	-	-
Hypervigilance subscale score	-	-	-	9	3.8	2.9	-	-
IES-R score <24	-	-	-	7	78	13.9	-	-
PTSD clinical concern (≥24)	-	-	-	2	22	13.9	-	-
IES-R score <33	-	-	-	8	89	10.5	-	-
PTSD probable diagnosis (≥33)	-	-	-	1	11	10.5	-	-

*SD* standard deviation

### Postvention protocol outcome indicators: posttraumatic growth and PTSD

Mean posttraumatic growth score among residents reporting patient suicide was 18 (SD = 7.3), which is considered “low” growth per the literature (Table 4). Mean posttraumatic growth scores were 16.5 (SD = 7.5) when patient suicide was in the last year and 21.0 (SD = 7.0) when patient suicide was 1–2 years ago. Though the descriptive trend suggests posttraumatic growth increased over time, these scores were not significantly different (*p* > 0.05). Scaled subscale scores show mean personal strength was highest (mean = 2.0, SD = 1.3). Posttraumatic growth among residents in this study was comparable to levels reported in another study of physicians who treat trauma victims (mean = 19.2, SD = 19.1) [31].

Mean PTSD score was 19.2 (SD = 10.7) among residents reporting patient suicide which is generally considered “low.” Approximately, 22% (*n* = 2) were at risk for PTSD, and 11% (*n* = 1) met criteria for PTSD diagnosis. Residents at risk for PTSD reported patient suicide in the last year. The proportion of residents in this study at risk for PTSD was comparable to proportions reported in other studies of inpatient nurses (13.7%) and French psychiatry trainees (16.8%) following patient suicide (*p* > 0.05, Table 4) [8, 32].

Posttraumatic growth was positively correlated with the self-determination subscale of work empowerment (*r =* 0.79, *p* = 0.01), but not associated with work empowerment or its other subscales, burnout, and general health measures. Mean PTSD score was not correlated with occupational health or general health measures (*p >* 0.05, results not shown).

### Resident feedback

Five out of nine residents (56%) indicated “no” to questions regarding whether the postvention protocol needed improvement. Three residents (33%) provided specific feedback. One resident shared that they experienced a second patient suicide at another program site and the postvention protocol had not been implemented in this instance. Another resident emphasized the importance of work modifications following the event:

The first few days after the event I did have a strong grieving response, and it would have been helpful to discuss options for temporarily alleviating other responsibilities to make that period more manageable.

Two residents recommended ongoing support following the postvention protocol. One resident stated that additional check-ins and group support throughout the year would be helpful. Another resident described providing peer support as a potential coping strategy:

I often process things by being able to mentor and share what I learned with others. If it became necessary and others faced a similar challenge, I would gain from being able to support them through it.

## Discussion

In 2016, the Joint Commission Advisory Group classified patient suicide as a sentinel event in psychiatry hospitals where residents typically work. Despite interventions implemented to predict and prevent suicide, the national US suicide rate increased by 33% from 1999 and inpatient suicides did not decrease [33]. Smith and colleagues argue that a “zero suicide” policy in hospitals promotes “dysregulation” and “maladaptive” reactions from clinicians when patient suicide occurs in these health systems [1]. In the context of increased adverse events and regulatory scrutiny, the postvention protocol was implemented in 2018 to address trainee distress following patient suicide. This is the first study we know of to evaluate a postvention protocol among psychiatry trainees.

Overall, 1 in 5 residents reported patient suicide at a psychiatry residency program located in the second largest city in the USA. Residents a part of this study see patients of high acuity in a variety of settings. The prevalence of patient suicide in our study shows that residents confront this occupational tragedy at high rates and at all levels of training. Interns report patient suicide despite their extended time on non-psychiatric services and short exposure as physicians. Senior residents who take care of less acute patients in clinic are not immune. One out of three fourth year residents reported patient suicide including a few months before graduation. Our findings show that equal proportions of residents report patient suicide during their junior and senior years of training. This is important when considering contextual factors that may contribute to the psychological impact of patient suicide across training levels. Junior residents treat more acutely ill patients in the hospital. They are also likely to experience patient suicide after discharging a patient deemed lower risk for suicide experiencing guilt and shock following the event. Senior residents may perceive their patients as more stable and themselves, more seasoned and by attribution bias believe they are less likely to encounter patient suicide. They are also susceptible to isolation in the outpatient setting where patient care is one-on-one, not team-based.

Process indicator results show that postvention protocol procedures occurred a majority of the time and at higher rates than reported in other studies [10]. That said, our results indicate that residents may need more prompting to seek self-care such as mental health services and work modifications. Since the culture of medical training can perpetuate self-management of one’s distress, residents may lack immediate insight regarding their needs and/or feel uncomfortable asking for what they need following an adverse event. Initially, the postvention protocol was designed for residents to opt-in to self-care services in order to maintain their autonomy and sense of agency following a destabilizing professional event. Rather than expecting an acutely traumatized resident to have the insight and foresight to “opt in” to support services though, it may be helpful for programs to closely follow up with residents reassessing their needs collaboratively. A formal write-up of the postvention protocol could also be helpful for trainees to refer to later on as they emerge from the acute grief of losing a patient. In general, increased formalization, discussion, and dissemination of a program’s postvention protocol may facilitate reducing stigma associated with patient suicide and stigma related to physicians receiving help. This program modified its online policies and procedures manual to include the postvention protocol schematic with supports and references. The program also initiated a postvention protocol information session during intern orientation.

Outcome indicator results suggest that postvention protocol-related supports were more impactful for residents than other forms of support such as speaking with a friend not in medicine. In addition, residents reported support from co-residents a part of and not a part of their patient’s care as helpful. These findings highlight the importance of peer support following patient suicide. If residents share their experiences and model vulnerability among their peers, the sense of isolation common after patient suicide may decrease. If a tradition such as this arises in which residents are available to each other following patient suicide, a peer-led support model may help normalize destructive thought patterns affected residents easily fall into. Residents described peer support in the form of frequent check-ins and pager coverage as “meaningful” and “caring.” Process groups received mixed reviews. One resident described a process group organized soon after their adverse event as “profoundly meaningful” and contributory to their healing process. Another resident found their process group “destabilizing” stating it took place in the middle of the work day and returning to work immediately afterwards was difficult. Study findings illustrating work empowerment, burnout, mental health risk, and quality of life did not differ among residents who participated in the postvention protocol compared to residents who had not experienced patient suicide suggests the protocol may reduce the negative impact of patient suicide among trainees. This is the first study we know of to examine occupational health and general health measures among residents who had underwent a postvention protocol against any comparison group.

Study results on posttraumatic growth highlight a potential mechanism by which residents positively cope with patient suicide. While posttraumatic stress symptoms following patient suicide have been documented in the literature, no previous study has examined posttraumatic growth [20]. In this study, residents reported enhanced personal strength following patient suicide and posttraumatic growth was correlated with greater work autonomy. Similarly, a study of pediatric hematology-oncology physicians showed that positive posttrauma changes following patient deaths were associated with increased professional self-esteem [34]. These findings bring up questions regarding the optimal context for promoting posttraumatic growth after patient suicide. Posttraumatic growth is observed to have a curvilinear relationship with posttraumatic stress in which both occur simultaneously while one navigates trauma [35]. By extension, do other forms of trauma such as moral distress influence trainees’ growth and stress responses following patient suicide [36]? Moral distress is defined as when one’s actions conflict with their values; and moral injury, the erosion of one’s moral conscience. Perceived personal and structural failures following patient suicide have the potential to cause moral injury. Moral injury may occur when a resident learns a patient committed suicide while incarcerated after having cleared them in the emergency room and returned them to police custody, or following discharge after a resident performed a thorough suicide risk assessment and safety plan with the patient. Therefore, other forms of trauma among trainees such as moral injury may be worthwhile to address in postvention protocols to enhance trainee growth.

The current study has several limitations. The cross-sectional study design prohibits causal inferences from being drawn from associations reported. The small study sample limits generalizability of findings and increases the type II error rate for non-significant findings. Evaluation of the postvention protocol was limited by the lack of an appropriate control group. Therefore, we are unable to delineate the impact of the postvention protocol from the experience of patient suicide alone. Other limitations include the use of self-report measures, retrospective reporting of PTSD symptoms, limited information on frequency, and timing of adverse event relative to outcomes examined. Study strengths include the high study response rate and use of multiple validated measures related to the impact of patient suicide among physicians, not previously examined.

In sum, the study findings inform standardization of postvention protocols among psychiatry training programs. In medicine, numerous approaches to addressing patient death have emerged such as debriefings, death rounds, quality care rounds, and crisis support teams along with postvention protocols [8, 15, 30, 37–39]. This study’s evaluation of a postvention protocol suggests it may be effective at mitigating the psychological and professional ramifications of patient suicide. The findings challenge the assumption that discussion of patient suicide soon after the event is harmful. We found that a number of different interactions and techniques delivered at the program level may facilitate coping following patient suicide. Some guiding principles are first and foremost to decrease the feelings of isolation among resident physicians. Second is to help trainees understand that bad patient outcomes are not personal failures. Third, a program’s training community should respond with kindness and support to this professional tragedy, even when it takes more work and attention. Residents should be met with in a caring and vulnerable manner ushering them to learn and grow from suicide.
